# Recurrence of COVID-19 associated with reduced T-cell responses in a monozygotic twin pair

**DOI:** 10.1098/rsob.210240

**Published:** 2022-02-02

**Authors:** Mateus V. de Castro, Keity S. Santos, Juliana S. Apostolico, Edgar R. Fernandes, Rafael R. Almeida, Gabriel Levin, Jhosiene Y. Magawa, João Paulo S. Nunes, Mirian Bruni, Marcio M. Yamamoto, Ariane C. Lima, Monize V. R. Silva, Larissa R. B. Matos, Vivian R. Coria, Erick C. Castelli, Marilia O. Scliar, Andreia Kuramoto, Fernanda R. Bruno, Lucas C. Jacintho, Kelly Nunes, Jaqueline Y. T. Wang, Veronica P. Coelho, Miguel Mitne Neto, Rui M. B. Maciel, Michel S. Naslavsky, Maria Rita Passos-Bueno, Silvia B. Boscardin, Daniela S. Rosa, Jorge Kalil, Mayana Zatz, Edecio Cunha-Neto

**Affiliations:** ^1^ Human Genome and Stem Cell Research Center (HUG-CELL), Biosciences Institute, Universidade de São Paulo, São Paulo, SP, Brazil; ^2^ Laboratory of Immunology, Heart Institute (InCor), Hospital das Clínicas da Faculdade de Medicina da Universidade de São Paulo, (HCFMUSP), São Paulo, SP, Brazil; ^3^ Institute for Investigation in Immunology—Instituto Nacional de Ciência e Tecnologia—iii-INCT, São Paulo, SP, Brazil; ^4^ Division of Clinical Immunology and Allergy, Department of Medicine, Faculdade de Medicina da Universidade de São Paulo (FMUSP), São Paulo SP, Brazil; ^5^ Department of Microbiology, Immunology and Parasitology, Universidade Federal de São Paulo (UNIFESP/EPM), São Paulo, SP, Brazil; ^6^ Department of Parasitology, Biosciences Institute, Universidade de São Paulo, São Paulo, SP, Brazil; ^7^ School of Medicine, Universidade Estadual Paulista (UNESP), Botucatu, SP, Brazil; ^8^ Fleury Laboratory, São Paulo, SP, Brazil

**Keywords:** COVID-19, twins, severe acute respiratory distress syndrome coronavirus 2, T cell, recurrence, immunity

## Abstract

Recurrence of COVID-19 in recovered patients has been increasingly reported. However, the immune mechanisms behind the recurrence have not been thoroughly investigated. The presence of neutralizing antibodies (nAbs) in recurrence/reinfection cases suggests that other types of immune response are involved in protection against recurrence. Here, we investigated the innate type I/III interferon (IFN) response, binding and nAb assays and T-cell responses to severe acute respiratory distress syndrome coronavirus 2 (SARS-CoV-2) with IFN gamma (IFN*γ*) enzyme-linked spot assay (ELISPOT) in three pairs of young adult monozygotic (MZ) twins with previous confirmed COVID-19, one of them presenting a severe recurrence four months after the initial infection. Twin studies have been of paramount importance to comprehend the immunogenetics of infectious diseases. Each MZ twin pair was previously exposed to SARS-CoV-2, as seen by clinical reports. The six individuals presented similar overall recovered immune responses except for the recurrence case, who presented a drastically reduced number of recognized SARS-CoV-2 T-cell epitopes on ELISPOT as compared to her twin sister and the other twin pairs. Our results suggest that the lack of a broad T-cell response to initial infection may have led to recurrence, emphasizing that an effective SARS-CoV-2-specific T-cell immune response is key for complete viral control and avoidance of clinical recurrence of COVID-19.

## Graphical abstract

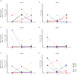

## 1. Background

Clinical recurrence of PCR-confirmed COVID-19 in adults with previous infection has been increasingly reported [1]. This has been attributed to a viral relapse in a host that failed to completely eradicate the virus or to reinfection with a different viral genome [2,3]. The involvement of innate/Type I/III IFN response as well as the specific antibody and T-cell responses is well established in the protection against severe acute respiratory distress syndrome coronavirus 2 (SARS-CoV-2) [4]. However, the immune mechanisms underlying reinfection/virus relapse are mostly unexplored. In this context, investigation of anti-SARS-CoV-2 immunity in clinical recurrence/reinfection cases has only been directed to the humoral response [5]. Since COVID-19 reinfection can occur both even in the presence of significant neutralizing antibody (nAb) titres [6], additional immune responses apart from humoral response may be involved in controlling reinfection and recurrence. The investigation of COVID-19 reinfection/recurrence can thus provide key information regarding immune protection mechanisms and guide vaccine development [6].

Studies with monozygotic (MZ) twins regarding viral infections have been a valuable source since they allow deep analysis of the environmental and host influences from different infectious agents [7]. In this article, we present a comprehensive assessment of innate and adaptive immunity in three pairs of recovered young adult MZ twins with confirmed COVID-19 and one recurrence case requiring admission to an intensive care unit.

## 2. Methods

### 2.1. Participants recruitment

Three pairs of COVID-19 recovered young adult MZ twins from São Paulo (the most populous city in Brazil) who were living together were recruited at the Human Genome and Stem Cell Research Center (HUG-CELL): a pair of MZ twin sisters who acquired mild COVID-19 in early 2020 where one, a healthcare worker, displayed severe clinical recurrence of COVID-19 four months after initial infection (ID 01 and ID 02); a second pair of MZ twin brothers with concordant asymptomatic infection (ID 03 and ID 04); and a third pair of MZ twin brothers with discordant symptomatic infection (ID 05 and ID 06). Baseline characteristics of the six twins are shown in table 1.

**Table 1.** Demographic, clinical data and human leukocyte antigen (HLA) information of the participants. Each monozygotic twin pair shared the same bedroom and was previously exposed to SARS-CoV-2 at home, according to clinical reports.

	MZ twin pair 1		MZ twin pair 2		MZ twin pair 3	
general information						
ID	01	02	03	04	05	06
sex	F	F	M	M	M	M
age	26	26	18	18	23	23
occupation	dentist	lawyer	student	student	student	student
COVID-19-related events						
first exposure to SARS-CoV-2	March 2020	March 2020	June 2020	June 2020	April 2020	April 2020
symptoms	flu-like illness^a^, adenomegaly, anosmia	flu-like illness^a^ with anosmia	asymptomatic (household exposure to symptomatic PCR-positive parent)	asymptomatic (household exposure to symptomatic PCR-positive parent)	flu-like illness^a^ with anosmia	asymptomatic (household exposure to symptomatic PCR-positive brother)
first positive SARS-CoV-2 IgG	16 May 2020 MAGLUMI 2019 nCoV IgG (21 U)	16 May 2020 MAGLUMI 2019 nCoV IgG (15 U)	27 July 2020 ELISA IgG anti-spike (figure 2*b*)	27 July 2020 ELISA IgG anti-spike (figure 2*b*)	28 July 2020 ELISA IgG anti-spike (figure 2*c*)	28 July 2020 ELISA IgG anti-spike (figure 2*c*)
COVID-19 recurrence	29 Jun 2020 positive SARS-CoV-2 PCR (4 months post original infection): flu-like illness^a^, diarrhoea, coughing 6 July hospitalization desaturation Pulmonary impairment 7 July ICU admission 16 Jul hospital discharge	—	—	—	—	—
first blood draw^b^	25 Aug 2020	25 Aug 2020	27 July 2020	27 July 2020	28 July 2020	28 July 2020
second blood draw^c^	10 Nov 2020	10 Nov 2020	20 Jan 2021	20 Jan 2021	21 Jan 2021	21 Jan 2021
HLA						
HLA-A and	A * 02:01:01; A * 32:01:01		A * 03:01:01; A * 23:01:01		A * 11:01:01; A * 31:01:02	
HLA-B and	B * 51:01:01; B * 08:01:01		B * 07:02:01; B * 35:03:01		B * 18:01:01; B * 38:01:01	
HLA-C and	C * 07:01:01; C * 14:02:01		C * 07:02:01; C * 12:03:01		C * 07:01:01; C * 12:03:01	
HLA-DRB1	DRB1 * 03:01:01; DRB1 * 11:03:01		DRB1 * 14:54:01; DRB1 * 09:01:02		DRB1 * 04:03; DRB1 * 13:01:01	

^a^Flu-like illness: fever, headache, malaise, fatigue.

^b^Type I/III innate IFN response, anti-SARS-CoV-2 IgG IgA and IgM binding and neutralizing antibodies, anti-endemic coronavirus antibodies.

^c^IFN-gamma ELISPOT T-cell response.

### 2.2. Sample collection

Blood samples were collected for global immune profiling at two instances post infection. On the first blood draw, we assessed SARS-CoV-2 IgG, IgA and IgM against SARS-CoV-2 spike (S), receptor-binding domain (RBD) and nucleocapsid protein (NP), nAbs and antibodies against the RBD region of human endemic coronaviruses, at least four weeks after initial COVID-19 diagnosis and then in January 2021. Twins were followed up for 10 months after the first blood draw. Samples were taken in vacutainer tubes with sodium heparin (BD Biosciences, USA, catalogue no. 367874) to obtain peripheral blood mononuclear cells (PBMCs); tubes without additives to obtain serum (BD Biosciences, USA, catalogue no. 366703) and tubes with ethylene diamine tripotassium (BD Biosciences, USA, catalogue no. 360057) to obtain plasma and for DNA extraction. Plasma and serum were obtained by centrifugation for 10 min at 2000*g* at room temperature within 30 min after blood draw. After this, the supernatant was transferred in aliquots of 1.5 ml into cryo vials (Corning, USA, catalogue no. 430487), and samples were transferred to a –80°C freezer until the moment of use. PBMCs were obtained by centrifugation of PBS-diluted 1 : 1 (Thermo Fisher Scientific, EUA, catalogue no. 10010031) blood samples in a leucosep tube (Greiner Bio-One, Austria, catalogue no. 163290) over a Ficoll-Paque (GE Healthcare Biosciences, USA, catalogue no. 17-5442-03) gradient following the manufacturer's instructions. The isolated PBMCs were stored in liquid nitrogen in solution of fetal bovine serum (FBS) (Sigma-Aldrich, USA, catalogue no. F4135) complemented with 10% dimethyl sulfoxide (Sigma-Aldrich, USA, catalogue no. D2650) until use.

### 2.3. Immunological assays

#### 2.3.1. Type I/III IFN innate immune response

Cryopreserved PBMCs were thawed and stimulated with 1 µg/ml of Poly I:C HMW (Invivogen, USA, catalogue no. tlrl-pic-5) for 1, 4 and 8 h. Negative controls were incubated with R10 medium alone. Total RNA was extracted using the RNeasy Mini kit (Qiagen, Germany, catalogue no. 74106), and cDNA was prepared using the Superscript II Reverse Transcriptase (Thermofisher Fisher Scientific, USA, catalogue no. 18064014), according to the manufacturer's instructions. Real-time PCR was performed using the Power SYBR Green Master Mix (Thermo Fisher Scientific, USA, catalogue no. 4368706) on a QuantStudio 12 K flex (Applied Biosystems, USA, catalogue no. 4471087). The cycling programme was used as follows: 95°C for 15 min; 40 cycles of 95°C for 15 and 60°C for 1 min. Primers used are listed in table 2.

**Table 2.** Primers used for reverse transcription polymerase chain reaction (RT-PCR) to assess the innate immune response.

RT-PCR primers		
gene	forward 5′ – 3'	reverse 5′ – 3'
*IFNA2*	TCGTATGCCAGCTCACCTTT	TCGTGTCATGGTCATAGCAGAA
*IFNB1*	ACGCCGCATTGACCATCTAT	GTCTCATTCCAGCCAGTGCT
*IRF7*	CTTCGTGATGCTGCGGGATA	TTCTCGCCAGCACAGCTC
*IFIT3*	AAGAACAAATCAGCCTGGTCAC	GACCTCACTCATGACTGCCC
*IFITM1*	GCCAAGTGCCTGAACATCTG	TGTCACAGAGCCGAATACCAG
*IFNL2*	TCCCAGACAGAGCTCAAAACT	CAGTCCCCTCTTCTGGATCTC
*IFNL3*	ACGCGAGACCTGAATTGTGT	TCAGGTTGCATGACTGGCG
*GAPDH*	CTCTGCTCCTCCTGTTCGAC	ATGGTGTCTGAGCGATGTGG

#### 2.3.2. Humoral immune response

SARS-CoV-2 IgG and IgM were initially detected by the clinical laboratory using the chemiluminescence immunoassay MAGLUMI 2019-nCoV IgM/IgG assay (Shenzhen New Industries Biomedical Engineering Co., Ltd, China, catalogue no: 130219018M). Enzyme-linked immunosorbent assay (ELISA) was performed using 96-well high-binding half-area polystyrene plates coated overnight at 4°C with 4 µg ml^−1^ of spike protein, 2 µg ml^−1^ NP (kindly provided by Dr Ricardo Gazzinelli, UFMG) or 0.8 µg ml^−1^ of the RBD domain from human endemic coronaviruses HKU-1, OC43, NL63 and 229E, all expressed in HEK293T cells. Plasmids encoding endemic coronavirus RBD domains are described in [8]. Patients' plasma samples were incubated at 56°C for 30 min, diluted 1 : 100 and run in triplicates. In short, 50 µl of diluted sera were incubated at 37°C for 45 min. Peroxidase-conjugated goat anti-human IgG (BD Pharmingen,USA), anti-human IgA (KPL, USA) or anti-human IgM (Sigma, USA) secondary antibody conjugates were diluted 1 : 10 000 and incubated at 37°C for 30 min. Values were determined as optical density minus blank, and cutoff was determined as blank+ 3× s.d. Besides, the pseudovirus neutralization assay was performed exactly as described previously [9]. HT1080 expressing ACE2 cells (HT1080/ACE2) and plasmids HIV-1NLΔEnv-NanoLuc and pSARS-CoV-2-SΔ19 were kindly provided by Dr Paul D. Bieniasz (The Rockefeller University). Briefly, 104 HT1080/ACE2 were plated in 96-well plates and maintained at 37°C, 5% CO_2_ for 24 h. The pseudovirus was incubated in duplicate with serial dilutions of the samples for 1 h at 37°C. After 48 h of incubation at 5% CO_2_ at 37°C, wells were washed, and cells were lysed with Lysis Buffer (Promega, USA, catalogue no E2661). Luciferase substrate (Promega, USA, catalogue no E1500) was added to each well, and the plate read at a GlowMax luminometer (Promega, USA, catalogue no GM2000). Fifty per cent inhibitory dilution was calculated using Prism software (v. 7.0, GraphPad) after subtraction of the background RLUs in the control wells (cells only).

#### 2.3.3. IFN*γ* ELISPOT assay

SARS-CoV2 specific T-cell responses were assessed using human ex vivo IFN*γ* enzyme-linked spot assay (ELISPOT) against a set of 20 CD4+ and 26 CD8+ T-cell epitopes from 13 distinct SARS-CoV-2 proteins with high HLA allelic population coverage in isolated PBMCs of each volunteer, three to four months after the COVID-19 episode or hospital discharge in the recurrence case. We identified and synthesized the CD4+ T-cell epitopes by scanning the whole proteome in SARS-CoV-2 reference genome (RefSeq: NC_045512.2) using the promiscuous HLA-DR binding peptide approach [10]. The chosen CD8+ T-cell epitopes were known to bind stably (www.immunitrack.com) or to be directly recognized [11] in the context of the 10 most frequent HLA class I alleles. The world population coverage of HLAs predicted to bind to the 20 CD4+ T-cell epitopes and 26 CD8+ T-cell epitopes was 99.6% and 94%, respectively, according to the IEDB epitope database [12]. Peptide sequences are listed in table 3. Cryopreserved PBMCs were thawed and rested overnight in R10 medium (RPMI supplemented with 10% of FBS, 2 mM l-glutamine, 1% v/v vitamin solution, 1 mM sodium pyruvate, 1% v/v non-essential amino acids, 50 U ml^−1^ penicillin/streptomycin and 5 × 105 M of 2-mercaptoethanol (Thermofisher, USA, catalogue no. 15070063) containing 30 U ml^−1^ of recombinant human IL-2 (ProleukinTM, Boehringer Ingelheim Pharma, Germany, catalogue no. PHC0023). Cells were seeded at 105 cells/well in MultiScreen MAIPS Filter Plates (Merck, USA, catalogue no. MAIPS4510) using coating and secondary anti-IFN*γ* antibodies (BD Biosciences). Incubation was performed for 18 h with synthetic peptides (5 ug ml^−1^; Genscript), medium alone or phorbol 12-myristate 13-acetate plus ionomycin (50 ng ml^−1^ and 1 ug ml^−1^, respectively) and developed with AEC substrate. Spots were counted using an AID ELISpot Reader System (Autoimmun Diagnostika GmbH, catalogue no ELR08IFL). The number of IFN*γ* producing cells/106 PBMC for each peptide was calculated after subtracting the values of control wells (R10 medium alone) for each subject. The cutoff value (105 IFN*γ* producing cells/106 PBMC) was established as the average + 3 s.d. of test results of the 46 peptides on cryopreserved PBMC from 19 pre-pandemic Brazilian healthy control subjects (data not shown).

**Table 3.** CD4+ and CD8+ T-cell epitopes used in ELISPOT assay.

protein	start–end	sequence
CD4+ T-cell epitopes		
spike	443–466	GNYNYLYRLFRKSNLKPFER
spike	334–352	FGEVFNATRFASVYA
spike	1086–1105	KAHFPREGVFVSNGTHWFVT
spike	503–522	VGYQPYRVVVLSFELLHAPA
spike	1009–1028	QLIRAAEIRASANLAATK
spike	896–915	IPFAMQMAYRFNGIGVTQNV
spike	747–763	TECSNLLLQYGSFCTQL
envelope	55–72	SFYVYSRVKNLNSSRVPD
membrane	43–62	NRFLYIIKLIFLWLLWPVTL
membrane	63–81	ACFVLAAVYRINWITGGIA
membrane	98–113	ASFRLFARTRSMWSFN
nucleocapsid	212–234	ALALLLLDRLNQLESKM
nucleocapsid	80–97	DQIGYYRRATRRIRGG
nucleocapsid	308–330	SAFFGMSRIGMEVTPSGTW
NSP3	3589–3613	TSLLVLVQSTQWSLF
ORF3a	26–40	SDFVRATATIPIQAS
ORF3a	118–137	INFVRIIMRLWLCWKCRSKN
ORF7a	105–120	AAIVFITLCFTLKRKT
ORF8	43–57	SKWYIRVGARKSAPL
ORF8	1–17	MKFLVFLGIITTVAAFH
CD8+ T-cell epitopes		
spike	89–97	GVYFASTEK
spike	269–277	YLQPRTFLL
spike	269–277	MIAQYTSAL
spike	691–699	SIIAYTMSL
spike	1220–1228	FIAGLIAIV
nucleocapsid	307–315	FAPSASAFF
nucleocapsid	219–227	LALLLLDRL
nucleocapsid	222–230	LLLDRLNQL
membrane	171–179	ATSRTLSYY
membrane	61–70	TLACFVLAAV
NSP3	1081–1089	YYKKDNSYF
NSP3	1374–1382	ASMPTTIAK
NSP3	1802–1810	AELAKNVSL
NSP3	686–694	TISLAGSYK
NSP3	887–895	GEAANFCAL
NSP5	219–227	FLNRFTTTL
NSP6	84–92	VYMPASWVM
NSP9	23–31	CTDDNALAY
RNA polymerase	253–261	AESHVDTDL
RNA polymerase	500–508	KSAGFPFNK
RNA polymerase	907–915	LTNDNTSRY
exonuclease	223–231	TYACWHHSI
exonuclease	232–240	GFDYVYNPF
exonuclease	288–296	KRVDWTIEY
exonuclease	487–495	HANEYRLYL
helicase	386–394	VVNARLRAK

### 2.4. Genomic assays

Whole-exome sequencing was performed on peripheral blood DNA in Illumina NovaSeq platform at HUG-CELL facilities. Sequencing data were analysed following bwa-mem, and GATK Best Practices workflow, quality control and annotation were performed as previously described [13]. HLA genes were realigned and called using HLA-mapper, which reduces mapping and calling errors [14].

## 3. Results and discussion

Here, we investigated the immunological profiles of three pairs of recovered young adult MZ twins, living together with confirmed COVID-19: (i) a pair of MZ twin sisters who acquired mild COVID-19 in March 2020 where one, a healthcare worker, displayed severe clinical recurrence of COVID-19 four months after initial infection (July 2020); (ii) a second pair of MZ twin brothers with concordant asymptomatic infection in June 2020; and (iii) a third pair of MZ twin brothers with discordant infection (one symptomatic and one asymptomatic) in April 2020. Demographical data, clinical manifestation of COVID-19, laboratory results and dates of specific immunological tests for all twin pairs are summarized in table 1 as well HLA-A, HLA-B, HLA-C and HLA-DRB1 alleles of each of the twin pairs.

**Figure 1.**
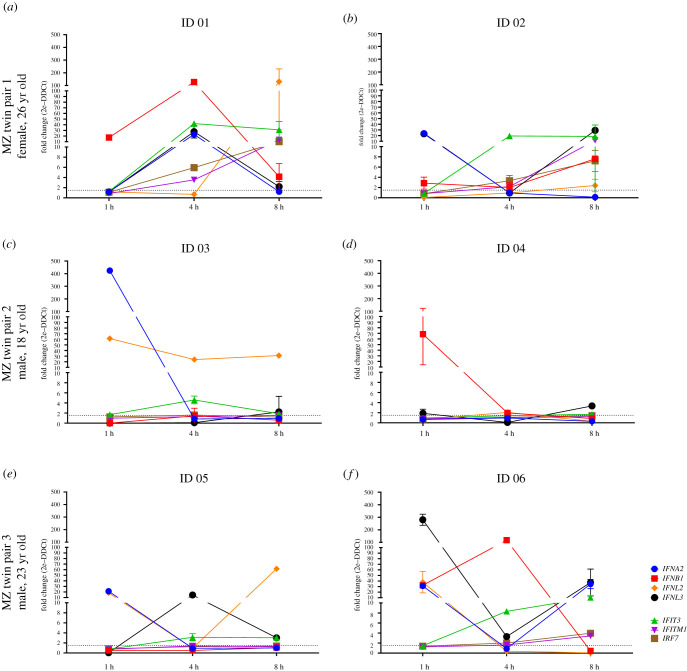
Early transcriptional type I/type III IFN innate immune response. PBMCs were stimulated with 1 µg ml^–1^ of double-stranded RNA Poly I:C for 1, 4 and 8 h. Total RNA was extracted for qPCR. Gene expression is relative to unstimulated cells. Expression kinetics of type I/type III genes after TLR3 stimulus for twin pair 1 (female): (*a*) ID 01 = severe clinical recurrence of COVID-19, (*b*) ID 02 = mild COVID-19 (non-recurrence); twin pair 2 (male): (*c*) ID 03 and (*d*) ID 04, asymptomatic infections; twin pair 3 (male): (*e*) ID 05 = mild COVID-19 and (*f*) ID 06 = asymptomatic infection. All subjects presented an early and strong (FC = 20 or higher) mRNA expression of at least one of the four type I/III IFN (*IFNA2, IFNB1, IFNL2* and *IFNL3*) at the earliest time point after Poly I:C stimulus.

Since innate type I/III IFN responses are the first line of cellular defence against RNA viruses, we evaluated the type I/III IFN production by PBMCs after toll-like receptor (TLR) stimulus (double-stranded RNA Poly I:C). Although there was heterogeneity in expression of the particular IFN or IFN-induced gene expression, all subjects presented an early and strong (FC = 20 or higher) mRNA expression of at least one of the four type I/III IFN (*IFNA2, IFNB1, IFNL2, IFNL3*) at the earliest time point after Poly I:C stimulus (figure 1). The finding that all six tested volunteers including the COVID-19 recurrence case presented an early and strong type I/III IFN response indicates that recurrence was not associated with failure in the innate IFN response.

**Figure 2.**
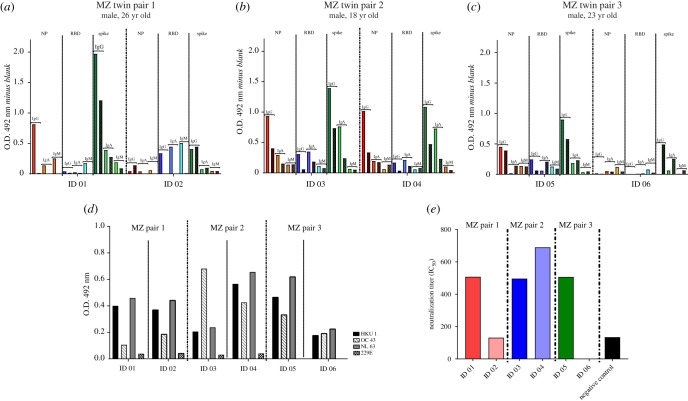
Humoral immune response profiles. (*a*–*c*) SARS-CoV-2-binding specific antibodies (IgG, IgA and IgM) against trimeric spike (S), the receptor-binding domain (RBD) and the nucleocapsid protein (NP). (*d*) IgG against RBDs of human endemic alpha- and beta-coronaviruses NL63, 229E, HKU1 and OC43. (*e*) SARS-CoV-2 neutralizing antibody activity using pseudotyped and chimeric viruses. Antibody responses for SARS-CoV-2 and endemic human coronaviruses were evaluated by ELISA. All subjects displayed IgG against the spike protein, confirming that all of them had been infected by SARS-CoV-2. Besides, the antibody response to endemic human coronaviruses was virtually identical in each twin pair, indicating no difference in exposure to these potentially cross-reactive viruses. Neutralizing antibodies (nAb) were detected in all of them except for ID 06 (asymptomatic infection).

To investigate the humoral immune response, we performed serological assays for SARS-CoV-2 IgA, IgG and IgM through ELISA for S, RBD and NP proteins (figure 2*a*–*c*). We observed that all MZ twins displayed IgG against the spike protein, confirming that all of them had been infected by SARS-CoV-2. However, detection of IgG-RBD and IgG-NP was variable among the MZ twins. As expected, the antibody titres against SARS-CoV-2 declined with time when comparing the two blood collections. On the other hand, IgG antibodies against the RBD of the four main circulating endemic coronaviruses, NL63, 229E, HKU1 and OC43, were similar for all six cases, displaying antibodies for three of four tested coronaviruses. The antibody profiles to human endemic coronaviruses were virtually identical in the MZ twin siblings (figure 2*d*).

In addition, nAbs titres were around 1 : 500 for the recurrent sister (ID 01), while the sister without recurrence (ID 02) presented titres similar to the negative control (figure 2*e*). For pair 2, nAb levels were similar to those of ID 01. On the other hand, the symptomatic brother from pair 3 (ID 05) displayed nAb titres around 1 : 500, while nAb titres similar to the negative control nAb were observed in the asymptomatic brother (ID 06). nAbs were detected in several cases of reinfection/recurrence [6], suggesting that responses beyond nAbs are important to control reinfection.

**Figure 3.**
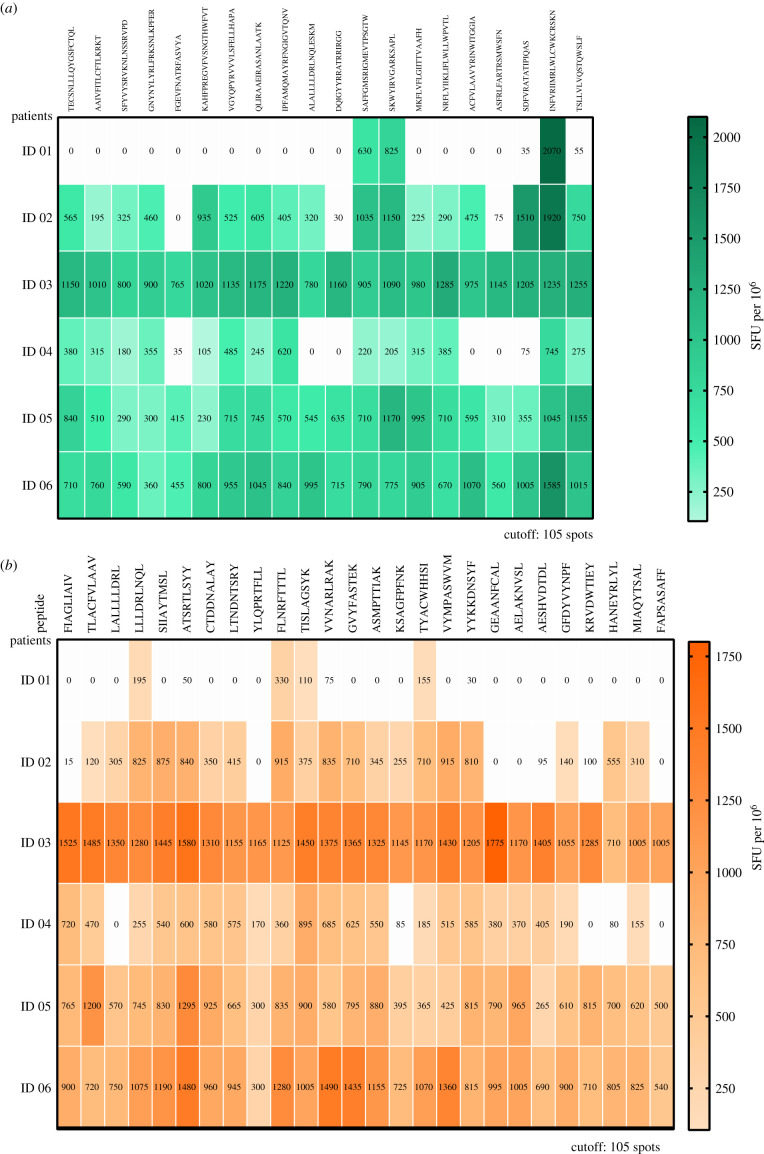
CD4+ and CD8+ T-cell responses to SARS-CoV-2 T-cell epitopes in isolated PBMCs, three to four months after COVID-19 episodes and the recurrence. T-cell responses were assessed using human ex vivo IFN*γ* ELISPOT against a set of 20 CD4+ (*a*) and 26 CD8+ (*b*) SARS-CoV-2 T-cell epitopes with high HLA allelic population coverage identified with bioinformatics tools. PBMC were stimulated with synthetic peptides for 18 h. All subjects recognized greater than 70% of CD4+ and CD8+ epitopes except the patient with severe recurring infection (ID 01) who recognized only 7 of 46 CD4+ and CD8+ T-cell epitopes (15%). Overall, the SARS-CoV-2 T-cell response is the only immune parameter that was substantially lower in the COVID-19 recurrence case (ID 01).

Finally, we assessed the T-cell responses to SARS-CoV-2 T-cell epitopes, by performing IFN*γ* ELISPOT assays (figure 3) on PBMC samples. We found that all recovered young adult MZ twins recognized greater than 70% of CD4+ and CD8+ epitopes except the patient with recurring infection (ID 01) who recognized only 7 of 46 CD4+ and CD8+ T-cell epitopes (15%), while her sibling recognized 36 CD4+ and CD8+ T-cell epitopes (78%; *p* < 0.0001, Fisher Exact Test). Thus, patient ID 01, who showed a COVID-19 recurrence, displayed a drastically reduced breadth (number of recognized epitopes) of both CD4+ and CD8+ SARS-CoV-2 T-cell epitopes as compared with her non-recurrent sibling and the others MZ twin pairs.

Interestingly, we found that the T-cell response is the only immune parameter that was substantially lower in the COVID-19 recurrence case ID 01. To our knowledge, this is the first report of T-cell immune responses in the context of COVID-19 recurrence and reinfection. Of note, there were also fluctuations in some other immune parameters within each MZ twin pair.

Our finding that the CD4+ and CD8+ T-cell response of the COVID-19 recurrence case had a drastically reduced breadth four months after hospital discharge is indicative of a low SARS-CoV-2-specific T-cell response. Given the importance of T-cell responses associated with COVID-19 infection [15], a dampened CD4+ T-cell response can have important consequences for many aspects of anti-SARS-COV-2 immunity. Asymptomatic and mild cases of COVID-19 are correlated with specific CD4+ and CD8+ T-cell responses, but not with IgG or nAb, suggesting that T cells are the primary effectors controlling a primary SARS-CoV-2 infection [4,16,17]. The dominant cytokine produced by virus specific CD4+ T cells is IFN*γ* with a Th1 profile, associated with antiviral activity. CD4+ T cells protect mice from lethal SARS-CoV infection [18], and Th1 CD4+ T cells are important to provide help for the cytotoxic CD8+ T responses crucial for clearance of viral infections. CD4+ T follicular helper cells contribute for B cell responses, and IL-22-producing T cells observed in COVID-19 are keys for maintenance of mucosal repair, particularly gut and lung epithelial cells [4].

It is unlikely that the reduced T-cell responses observed in ID 01 are due to the deficient HLA presentation to T cells, since her MZ twin ID 02 carrying the same HLA alleles displayed a broad recognition profile. Also, it is not likely that the contrasting T-cell responses observed between the two siblings is a result of previous exposure to cross-reacting coronaviruses [19] since their IgG profile against human endemic coronaviruses' RBD was nearly identical. Our findings are also illustrative that adaptive immune responses and clinical presentations of COVID-19 can be drastically different within a MZ twin pair. The observed diversity is consistent with the fact that T- and B-cell repertoire developments are somatic DNA rearrangement events likely to differ even among MZ twins.

In short, our results suggest that the failure in inducing a broad T-cell response might have enhanced susceptibility to COVID-19 recurrence in patient ID 01. Our data may support a prime role for T cells in protection against reinfection. Given the increased concern that SARS-CoV-2 variants escaping antibody neutralization could give rise to a massive raise in reinfection [20,21], our case stresses the importance of T-cell immune responses in protection against reinfection. This is in line with the reported lack of deleterious effect of virus variants in the cellular immune response [22]. Further investigation in a larger cohort can shed light on whether T-cell dysfunction is a common mechanism for recurrence of COVID-19.

## Data accessibility

This article has no additional data.
